# The antimicrobial protein, CAP37, is upregulated in pyramidal neurons during Alzheimer’s disease

**DOI:** 10.1007/s00418-015-1347-x

**Published:** 2015-07-14

**Authors:** Amanda J. Brock, Anne Kasus-Jacobi, Megan Lerner, Sreemathi Logan, Adekunle M. Adesina, H. Anne Pereira

**Affiliations:** Oklahoma Center for Neuroscience, University of Oklahoma Health Sciences Center, 1110 N. Stonewall Ave., CPB 255, Oklahoma City, OK 73117 USA; Department of Pharmaceutical Sciences, University of Oklahoma Health Sciences Center, 1110 N. Stonewall Ave., CPB 255, Oklahoma City, OK 73117 USA; Department of Surgery, University of Oklahoma Health Sciences Center, 1122 NE 13th St., ORB 350, Oklahoma City, OK 73117 USA; Department of Geriatrics, University of Oklahoma Health Sciences Center, 975 NE 10th St., BRC 1303, Oklahoma City, OK 73104 USA; Department of Cell Biology, University of Oklahoma Health Sciences Center, 1110 N. Stonewall Ave., CPB 329, Oklahoma City, OK USA; Department of Pathology, University of Oklahoma Health Sciences Center, 1110 N. Stonewall Ave., CPB 329, Oklahoma City, OK 73117 USA; Department of Pathology, Baylor College of Medicine, One Baylor Plaza, Rm 286A, Houston, TX 77030 USA

**Keywords:** Neuroinflammation, CAP37, Alzheimer’s disease, Neurons, Amyloid-beta, Microglia

## Abstract

Inflammation is a well-defined factor in Alzheimer’s disease (AD). There is a strong need to identify the molecules contributing to neuroinflammation so that therapies can be designed to prevent immune-mediated neurotoxicity. The cationic antimicrobial protein of 37 kDa (CAP37) is an inflammatory mediator constitutively expressed in neutrophils (PMNs). In addition to antibiotic activity, CAP37 exerts immunomodulatory effects on microglia. We hypothesize that CAP37 mediates the neuroinflammation associated with AD. However, PMNs are not customarily associated with the pathology of AD. This study was therefore designed to identify non-neutrophilic source(s) of CAP37 in brains of AD patients. Brain tissues from patients and age-matched controls were analyzed for CAP37 expression using immunohistochemistry (IHC). To determine factors that induce CAP37 in AD, HCN-1A primary human neurons were treated with tumor necrosis factor-alpha (TNF-α) or amyloid β_1−40_ (Aβ) and analyzed by IHC. Western blotting and quantitative reverse transcription polymerase chain reaction (qRT-PCR) were used to confirm CAP37 expression in neurons and brain tissues. IHC revealed CAP37 in cortical neurons in temporal and parietal lobes as well as CA3 and CA4 hippocampal neurons in patients with AD. CAP37 was found in more neurons in AD patients compared with age-matched controls. qRT-PCR and Western blotting showed an increase in CAP37 transcript and protein in the AD temporal lobe, a brain region that is highly impacted in AD. qRT-PCR observations confirmed CAP37 expression in neurons. TNF-α and Aβ increased neuronal expression of CAP37. These findings support our hypothesis that neuronal CAP37 may modulate the neuroinflammatory response in AD.

## Introduction

The cationic antimicrobial protein of molecular weight 37 kDa (CAP37) is an inflammatory mediator expressed constitutively in the azurophil granules of polymorphonuclear neutrophils (PMNs), and is considered an important component of the innate immune system (Pereira et al. 1990a; Griffith et al. 2013). Previous findings have shown increased CAP37 levels during inflammatory conditions such as sepsis and atherosclerosis (Lee et al. 2002; Pereira et al. 2003; Linder et al. 2009). CAP37 has potent antimicrobial activity (Pereira et al. 1993, 1995, 2006) and also exerts various regulatory functions in mammalian cells. Some of these functions include activation of protein kinase C (PKC) and upregulation of adhesion proteins on endothelial cells and corneal epithelial cells; contraction of endothelial cells; proliferation of smooth muscle cells; corneal epithelial wound healing; and chemotaxis of monocytes, microglia, smooth muscle cells, and corneal epithelial cells (Pereira et al. 1990a, 2003, 2004; Gautam et al. 2001; Gonzalez et al. 2004; Griffith et al. 2014).

CAP37 is induced in corneal epithelial cells, endothelial cells, and smooth muscle cells in response to cytokines, lipopolysaccharide (LPS), and infection (Pereira et al. 1996b; Lee et al. 2002; Ruan et al. 2002; Gonzalez et al. 2004). In addition, CAP37 expression has been found in endothelial cells of hippocampal vasculature in patients with Alzheimer’s disease (AD), while this expression was absent in age-matched controls (Pereira et al. 1996a). This finding is important, as the hippocampus is responsible for memory formation and is one of the main regions where AD pathology first manifests (Pereira et al. 1996a). Although CAP37 shares ~45, ~42, and ~32 % sequence homology with the serine proteases elastase, proteinase 3, and cathepsin G, respectively, CAP37 itself lacks serine protease activity due to the loss of 2 of 3 conserved residues of the catalytic triad (Pereira et al. 1990b). Elastase and proteinase-3 have been observed in various regions of the brain, including the hippocampus, cerebellum, and cerebral cortex (Davies et al. 1998), and elastase has been detected in murine microglial cells (Nakajima et al. 1992). However, the exact cell expression profile of these other proteases in the human brain is unknown. Expression of human β-defensin-1, another cationic antimicrobial peptide, has been reported within brain hippocampal astrocytes, neurons, and the choroid plexus and was increased in these regions in patients with AD (Williams et al. 2013). The role of these proteins in AD is unknown. However, since all are involved in innate immunity and defense, their expression in brain cells raises the question of whether any of these proteins might have a role in the low-grade chronic inflammatory response that occurs in neurodegenerative diseases such as AD (Wilson et al. 2002; Heneka et al. 2010; Hensley 2010; Grammas 2011; Eikelenboom et al. 2012).

A primary research focus of our laboratory is defining the mechanisms whereby CAP37 modulates neuroinflammation. As CAP37 is a potent activator of microglia, we hypothesized that CAP37 expressed within the brain parenchyma was one of the mediators of neuroinflammation in Alzheimer’s disease. Thus, in this study, we aimed to determine the CAP37 cellular expression and localization in brains from patients with Alzheimer’s disease. Our results indicate that CAP37 is expressed in neutrophils, the vascular endothelium, and neurons in specific brain regions from patients with AD. CAP37 transcript and protein levels are increased in patients with AD, and primary neurons can be induced to express CAP37 in response to tumor necrosis factor-alpha (TNF-α) and amyloid-beta (Aβ).

## Materials and methods

### Tissue specimens

Tissues from nine patients diagnosed with AD and nine respective age-matched controls were kindly provided by Dr. Eileen Bigio of the Department of Pathology, Northwestern University Feinberg School of Medicine, Alzheimer’s Disease Center, Neuropathology Core. All patients and age-matched controls were characterized for AD pathology using the Consortium to Establish a Registry for Alzheimer’s Disease (CERAD) plaque grades (A–C) and Braak tangles stages (I–VI) to determine the approximate disease stage. All AD tissue specimens used for IHC were given CERAD scores of C and Braak & Braak stage VI for tangles. Age-matched controls were given CERAD scores of A, B, or 0, and were Braak & Braak stages I–III or 0.

### Cell culture and treatment of HCN-1A neurons

We purchased HCN-1A, primary human cortical neuronal cells, from American Type Culture Collection (ATCC, Manassas, VA). Dulbecco’s modified Eagle’s medium (DMEM, ATCC) supplemented with 20 % bovine calf serum, 1 % antibiotic–antimycotic, and 1 % l-glutamine (Life Technologies, Grand Island, NY) was used to culture HCN-1A cells according to the ATCC recommendations. The HCN-1A cells were used in immunohistochemistry. During the course of our studies, ATCC discontinued the distribution of HCN-1A cells due to quick senescence of the cells and insufficient inventory. The HCN-1A cells were thus unavailable for use in other assays. HCN-1A cells were treated overnight (17 h) with either the 40 amino acid Aβ peptide (Aβ_1−40_, Bachem, Torrance, CA, stock solution in ultrapure water) at a concentration of 125 µg/ml or human recombinant tumor necrosis factor-alpha (TNF-α, Roche, Indianapolis, IN) at a concentration of 25 ng/ml. Control treatments included HCN-1A incubation with either inactive (reverse peptide order) Aβ peptide (Aβ_40−1_) (Bachem, stock solution in 10 % acetic acid) or vehicle alone (basal medium or basal medium containing an equivalent volume of 10 % acetic acid). Aβ was diluted in basal medium and incubated for 2 h at room temperature before administration, to allow the formation of toxic oligomers and fibrillar aggregates that are found in AD.

### Antisera

We used 7 μg/ml rabbit anti-CAP37 antiserum as described previously (Pereira et al. 1996a), a control of 7 μg/ml normal rabbit antiserum (Jackson ImmunoResearch Laboratories, West Grove, PA), an in-house made mouse monoclonal antibody to CAP37 (D5F10), and a mouse IgG1 isotype antibody was used as a control (Sigma-Aldrich, St. Louis, MO). D5F10 and the isotype control were diluted in Emerald antibody diluent (Cell Marque, Rocklin, CA) to a concentration of 4 μg/ml for IHC and were diluted in 2 % bovine serum albumin (BSA, Calbiochem, Billerica, MA) in Tris-buffered saline with 0.05 % Tween (TBST) blocking solution to a concentration of 0.2 μg/ml for Western blots. Rabbit polyclonal anti-Aβ (Cell Signaling, Danvers, MA) and rabbit polyclonal anti-phospho-tau (Santa Cruz, Dallas, TX) were diluted in Emerald antibody diluent to a concentration of 10 μg/ml to detect Aβ plaques and tau tangles, respectively. A rabbit IgG antibody was employed as an isotype control (Cell Signaling, Danvers, MA). We also used the following secondary antibodies: horseradish peroxidase (HRP)-conjugated donkey anti-mouse and donkey anti-rabbit IgGs diluted in TBST to concentrations of 0.04 μg/ml (Jackson ImmunoResearch Laboratories, West Grove, PA).

### Immunocytochemistry

HCN-1A cells were evenly seeded into 4-well LAB-TEK tissue culture chambers (NUNC, Inc., Naperville, IL) and incubated until confluency. Cells were then treated with either Aβ_1−40_, TNF-α, or serum-free DMEM as described above. Following treatment, cells were fixed with formol acetone and stained using the Vectastain ABC peroxidase system (Vector Laboratories, Burlingame, CA) as previously described (Pereira et al. 1996a). The rabbit anti-CAP37 and normal rabbit serum were added for 17 h. Images of stained neurons were taken at 400X magnification (Nikon TE2000, Nikon Instruments Inc., Melville, NY). Figures were created using Microsoft PowerPoint 2010.

### Immunohistochemistry

Formalin-fixed, paraffin-embedded brain tissues from patients with Alzheimer’s disease and age-matched controls were sectioned at a thickness of 5 μm. Sections were incubated in antigen retrieval solution (Tris buffer, pH 9) for 20 min in a rice steamer, followed by a 20-min cool down in distilled water. Staining was performed using reagents from the HiDef HRP kit (Cell Marque) according to the manufacturer’s instructions. Tissues were incubated with mouse anti-CAP37 (4 μg/ml) or the equivalent amount of mouse IgG1 isotype control for 60 min. Color was developed with 3,3′-diaminobenzidine (Cell Marque), and sections were counterstained with hematoxylin (American Master Tech, Lodi, CA). Images were examined, and photographs were taken using bright-field microscopy at 400X and 1000X magnifications (Nikon eclipse E200, Nikon Instruments Inc., Melville, NY). Figures were created using Microsoft PowerPoint 2010.

### Quantitative RT-PCR

Total RNA from primary human neurons and primary human astrocytes (Sciencell, Carlsbad, CA) and total RNA from temporal, frontal, and occipital lobe tissues of patients with AD and five donor pool normal controls (Biochain, Newark, CA) were purchased from their respective commercial vendors. AD patients included a 73-year-old male (frontal lobe RNA), 77-year-old male (occipital lobe RNA), 80-year-old male (temporal and frontal lobe RNA), 83-year-old male (temporal lobe RNA), 85-year-old female (occipital lobe RNA), and an 87-year-old male (temporal, frontal, and occipital lobe RNA) for a total of six patients analyzed with three utilized for each brain region. Pooled controls were all from males with ages ranging from 20 to 44 years. PCR-ready first-strand cDNA from peripheral blood leukocytes was obtained from Biochain. PCR-ready first-strand cDNA from human microglia was obtained from Sciencell. Primary neuron RNA (2 μg), primary astrocyte RNA (2 μg), AD patient RNA (3 μg), and normal control pooled RNA (3 μg) were converted to cDNA using the Qiagen RT^2^ First-Strand Kit (Qiagen Inc., Valencia, CA) in a final volume of 111 μL according to the manufacturer’s instructions. Amplification of cDNA was performed using RT^2^ SYBR Green mastermix (containing HotStart DNA Taq Polymerase), Qiagen RT^2^qPCR primers (*GAPDH*: #PPH00150F, PRTN3: #PPH07029A, *ELANE*: #PPH01057A, *AZU1*: #PPH01031A), and Solaris primers (*CTSG* #AX-005838-00-0100, *GAPDH*# AX-005838-00-0100), following the manufacturer’s instructions. For each 25-μL reaction, 1 μL of the prepared cDNA was mixed with 1 μL (0.4 μM) of respective primer, 12.5 μL of RT^2^ SYBR Green mastermix, and 10.5 μL of RNase-free water. All reactions were performed in triplicate. PCRs were performed using the MyiQ Single Color Real-Time PCR Detection System (Bio-Rad, Hercules, CA) beginning with a denaturation step (10 min at 95 °C) followed by 40 repeated cycles of annealing/extension: 15 s at 95 °C and 1 min at 60 °C. qRT-PCR was performed on primary neurons, primary microglia, primary astrocytes, and normal control pools twice and on AD patients and leukocytes once. The Δ*C*_t_ values were calculated to normalize each gene expression to glyceraldehyde 3-phosphate dehydrogenase (*GAPDH*). To calculate fold difference in mRNA expression relative to *GAPDH,* the equation [fold change = 2^(−Δ*C*t)^] was used. Figures were created using Prism software (GraphPad Software Inc., version 6, La Jolla, CA.)

### Western blot analysis

Total protein lysates from the temporal lobe of one patient with AD (75-year-old male), from the frontal lobe of one patient with AD (83-year-old male), and from the temporal (26-year-old male) and frontal (71-year-old male) lobes of normal controls were obtained from Biochain. According to the vendor, all tissues were obtained 4–6 h postmortem and were stored in liquid nitrogen before distribution. Human astrocyte and microglia lysates were derived from single healthy donors and obtained from Sciencell. The protein concentration of each sample was provided by the respective vendors. The protein concentration of total temporal and frontal lobe lysates was verified using the bicinchoninic acid (BCA) assay (Pierce, Rockford, IL). Purified CAP37 derived from human neutrophils (Athens Research Technology, Athens, Georgia) was also run as a marker for CAP37 migration. Total lysates from human astrocytes, human microglia, temporal lobes, frontal lobes (40 μg), PMNs (50 ng), and purified CAP37 (5 ng) were separated by a 12.5 % SDS-PAGE gel. Proteins were transferred to a nitrocellulose membrane (Whatman, Pittsburgh, PA) overnight, and the membranes were blocked in 2 % BSA in TBST. Membranes were probed with either mouse monoclonal anti-CAP37 (0.2 μg/ml) or the equivalent amount of mouse isotype control overnight at 4 °C. Blots were incubated with HRP-conjugated donkey anti-mouse secondary antibody (0.04 μg/ml; Jackson Laboratories) for 1 h at room temperature. Enhanced chemiluminescence (ECL) substrates (Pierce, Rockford, IL) were used to develop all blots. ImageJ software (National Institutes of Health [NIH], Bethesda, MD) was used to quantify mean band density. Figures were created using Microsoft PowerPoint 2010.

### Statistical analysis

Statistical analysis was performed using Prism software. Student’s unpaired *t* tests were used to analyze the *AZU1*, *ELANE*, and *PRTN3* mRNA expressions of individual patients with AD relative to the normal control pools. The mRNA expression values were calculated as described in the above methods and are represented as mean ± SEM, and *p* < 0.05 was considered statistically significant.

## Results

### Both parietal and temporal lobes from AD patients express CAP37

**Fig. 1:**
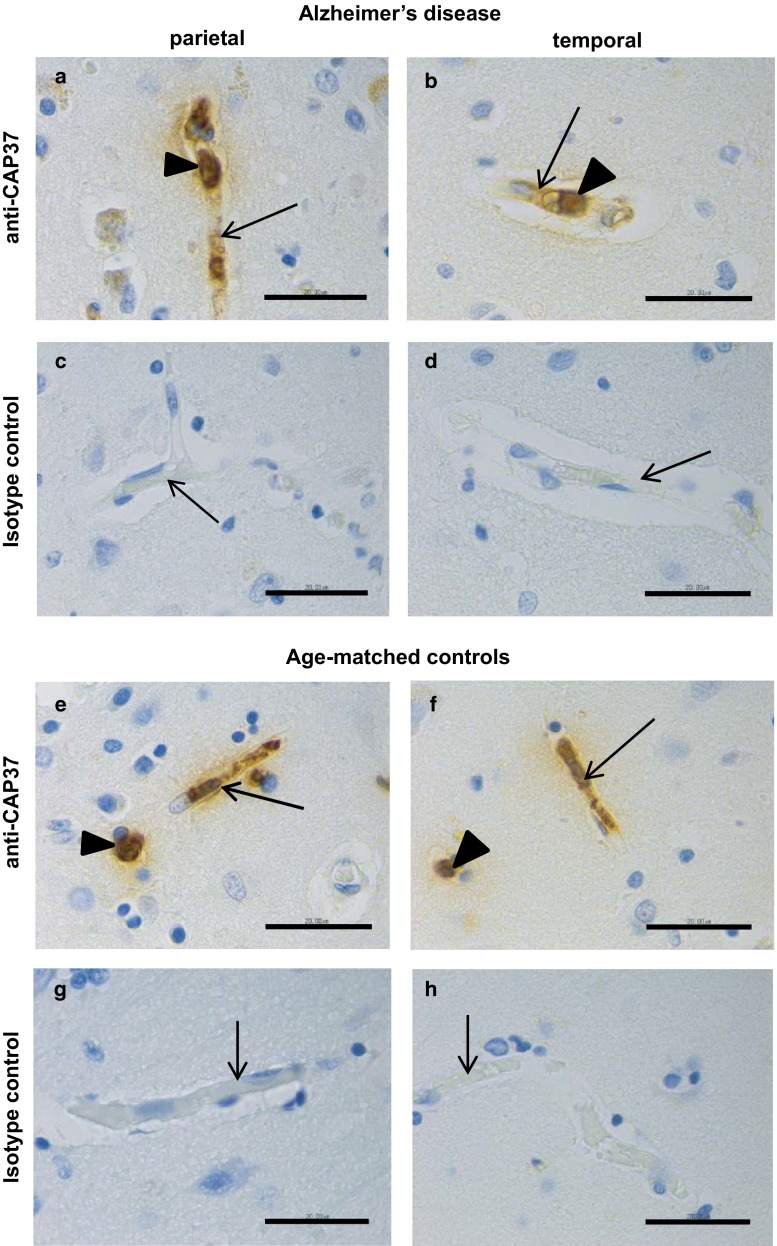
CAP37 is expressed in neutrophils and endothelial cells of parietal and temporal cortices. **a** Parietal lobe and **b** temporal lobe cortical tissues from an Alzheimer’s disease (AD) patient stained with anti-CAP37. **c** Parietal lobe and **d** temporal lobe cortical tissues from an AD patient stained with isotype control. **e** Parietal lobe and **f** temporal lobe cortical tissues from an age-matched control stained with anti-CAP37. **g** Parietal lobe and **h** temporal lobe cortical tissues from an age-matched control stained with isotype control. Neutrophils are indicated by *arrowheads*, and endothelial cells are represented by *arrows*. *Scale bars* 20 μm

CAP37 expression in the endothelial cells lining vessels of the hippocampus in patients with AD has been previously demonstrated (Pereira et al. 1996a). However, whether CAP37 was also expressed in additional brain regions from AD patients has not been studied. Therefore, parietal and temporal lobe tissues from patients with AD (*n* = 9) or age-matched normal controls (*n* = 9) were evaluated for CAP37 expression. Performing IHC using a novel monoclonal antibody specific for CAP37 detected expression of this immune modulator in the polymorphonuclear leukocytes (PMNs) and endothelial cells in both the parietal (Fig. 1a) and temporal lobes (Fig. 1b) of all AD patients and age-matched controls (Fig. 1e, f). Staining with isotype control antibody failed to detect PMNs and endothelial cells, indicating the specificity of staining for CAP37 in these two cell types (Fig. 1c, d, g, h). These assays confirmed the applicability of this monoclonal antibody for use in IHC on brain tissues since CAP37 is constitutively expressed in neutrophils and is also expressed in endothelial cells from other tissues.

### Cortical pyramidal neurons in temporal and parietal lobes are a novel cellular source of CAP37 expression and are a site of CAP37 upregulation in AD patients

**Fig. 2:**
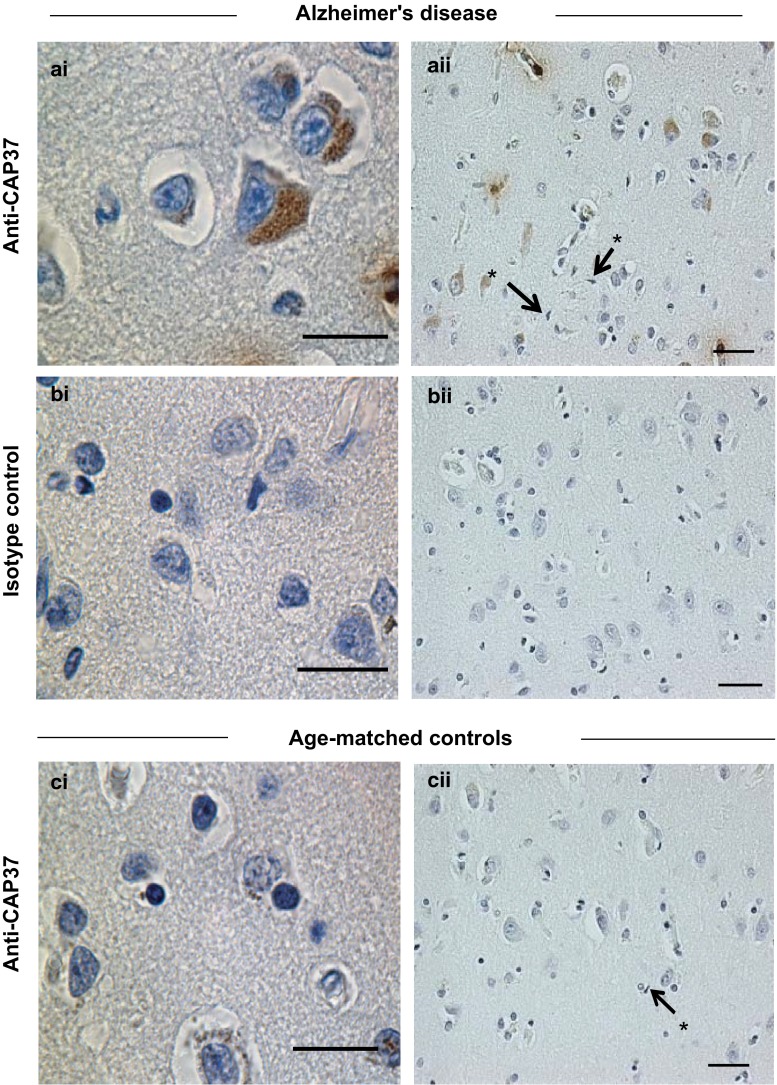
Temporal cortical neurons have increased CAP37 expression during AD. **ai** AD temporal cortical lobe tissue stained with monoclonal anti-CAP37 showing strong staining in the AD neuron cell bodies. CAP37 staining varied from localized staining in pyramidal layers 3 and 5 to a diffuse cortical distribution. **bi** AD temporal cortical lobe tissue stained with isotype control. **ci** Age-matched control brain stained with monoclonal anti-CAP37. Note the reduced staining in the neuron cell bodies. **aii**, **bii**, **cii** Lower magnification images of respective sections. *Asterisks* (*) with pointing *arrows* indicate microglial cells that lack CAP37. *Scale bars* **ai**, **bi**, and **ci**, 20 μm; **aii**, **bii**, and **cii**, 50 μm

**Fig. 3:**
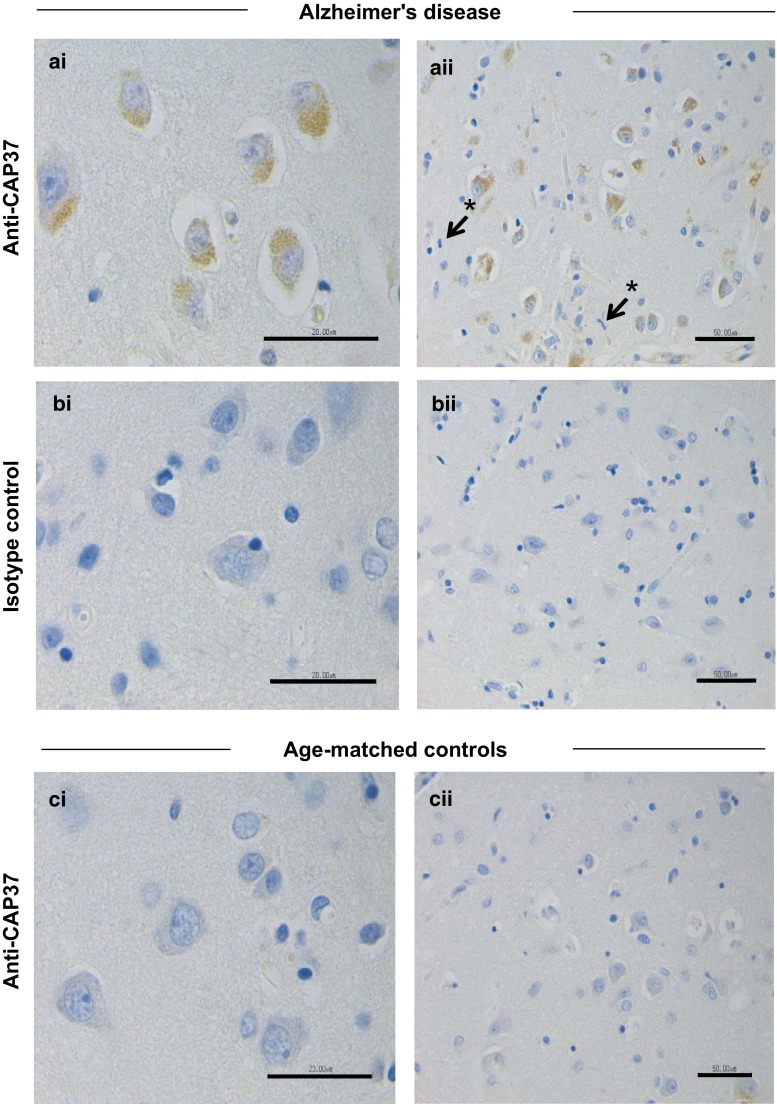
Parietal cortical neurons have increased CAP37 expression during AD. **ai** AD cortical brain tissue from parietal lobes stained with monoclonal anti-CAP37. Intense CAP37 staining is localized to the AD neuron cell bodies. CAP37 appeared in pyramidal layers 3 and 5 in some patients and showed a more diffuse cortical distribution in others **bi** AD parietal lobe tissues stained with isotype control. **ci** Age-matched control brain stained with monoclonal anti-CAP37 IgG. **aii**, **bii**, **cii** Lower magnification images of respective sections. *Asterisks* (*) with pointing arrows indicate microglial cells that lack CAP37. *Scale bars* **ai**, **bi**, and **ci**, 20 μm; **aii**, **bii**, **cii**, 50 μm

The temporal and parietal lobes of AD patients were then evaluated to identify additional CAP37 cellular sources. CAP37 was detected in the cytoplasm of cortical neurons in the temporal lobes of patients with AD (Fig. 2ai, aii). The pattern of CAP37 staining varied from localized in pyramidal layers 3 and 5 to a diffuse cortical distribution. We observed a predominant limitation to pyramidal layers 3 and 5 more often than a diffuse cortical pattern. However, a diffuse pattern was often associated with a more intense cytoplasmic staining on a per-patient basis. No temporal lobe staining was observed with the isotype control (Fig. 2bi, bii), indicating the CAP37 staining specificity in PMNs, endothelial cells, and neurons. CAP37 was observed in temporal lobe pyramidal layers 3 and 5 in some control subjects as well. However, fewer neurons were CAP37-positive in control tissues (Fig. 2ci, cii) than in tissues from patients with dementia. No CAP37 expression was observed in glial cells, including microglia (Fig. 2aii, cii). CAP37 staining in the parietal lobes of patients with AD and controls showed a similar pattern to that seen in the temporal lobes. Neurons within pyramidal layers 3 and 5 of the parietal cortex from AD patients were also positive for CAP37 (Fig. 3ai, aii). This was not observed with the isotype control (Fig. 3bi, bii). Again, some age-matched controls showed neuronal staining for CAP37 in the parietal lobes, but fewer control neurons stained positive than did AD neurons (Fig. 3ci, cii). No glial cells stained positive for CAP37 in the parietal lobe (Fig. 3aii).

### CAP37 is expressed in the CA3 and CA4 hippocampal neurons

**Fig. 4:**
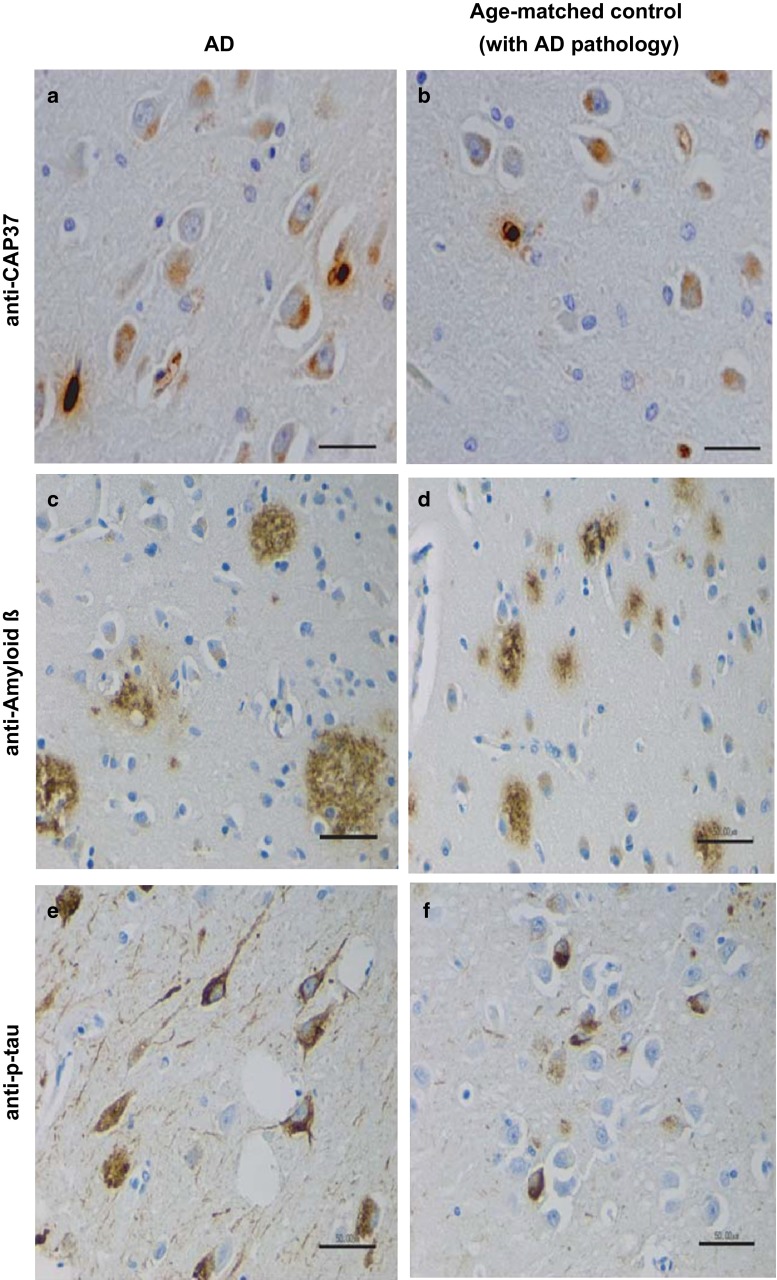
CAP37 is expressed in CA3 and CA4 hippocampal neurons. **a** AD and **b** age-matched control hippocampal tissue stained with anti-CAP37 showing staining in neuronal cell bodies. **c** AD patient and **d** age-matched control stained with anti-amyloid-*β* showing frequent neuritic plaques in both. **e** AD patient and **f** age-matched control stained with anti-phospho-tau (Thr 205) showing Braak stage VI tangles in patient and Braak stage II tangles in age-matched control. *Scale bars* **a** and **b**, 40 μm; **c**–**f**: 50 μm

The hippocampus was evaluated in AD patients using the CAP37 monoclonal antibody to determine additional CAP37 cellular sources other than sources previously revealed. IHC analysis on hippocampal sections from three patients with AD (Fig. 4a) and age-matched controls (Fig. 4b) revealed CAP37 expression in pyramidal neurons of the *Cornu Ammonis* (CA) regions 3 and 4. All three patients with AD were previously analyzed for AD pathology and characterized to have CERAD plaque scores of C and Braak and Braak stage VI for tangles. Our IHC staining also confirmed the high frequency of plaques (Fig. 4c) and tangles (Fig. 4e) in these patients. Although one of the age-matched controls showed no signs of neuritic plaques or tangles, the other two contained both plaques and tangles, with one also having frequent neuritic plaques (Fig. 4d) and Braak stage II for tangles (Fig. 4f). One age-matched control was, therefore, characterized as having low AD neuropathological change, while another was characterized as displaying intermediate AD pathological change.

Next, CAP37 association with either Aβ plaques or tau tangles was determined by IHC to analyze the localization patterns of CAP37, Aβ, and tau. Serial sections of the temporal cortex and hippocampus stained for CAP37, Aβ, and tau revealed that both Aβ and tau were more heavily distributed than the sporadic neuron staining observed for CAP37. Although CAP37, Aβ, and tau overlapped in some regions, there did not appear to be a strong correlation in localization as some regions with CAP37 did not show tangles or plaques, and many regions with plaques and tangles did not also contain CAP37 (data not shown).

### TNF-α and Aβ induced expression of CAP37 in human primary cortical neurons

**Fig. 5:**
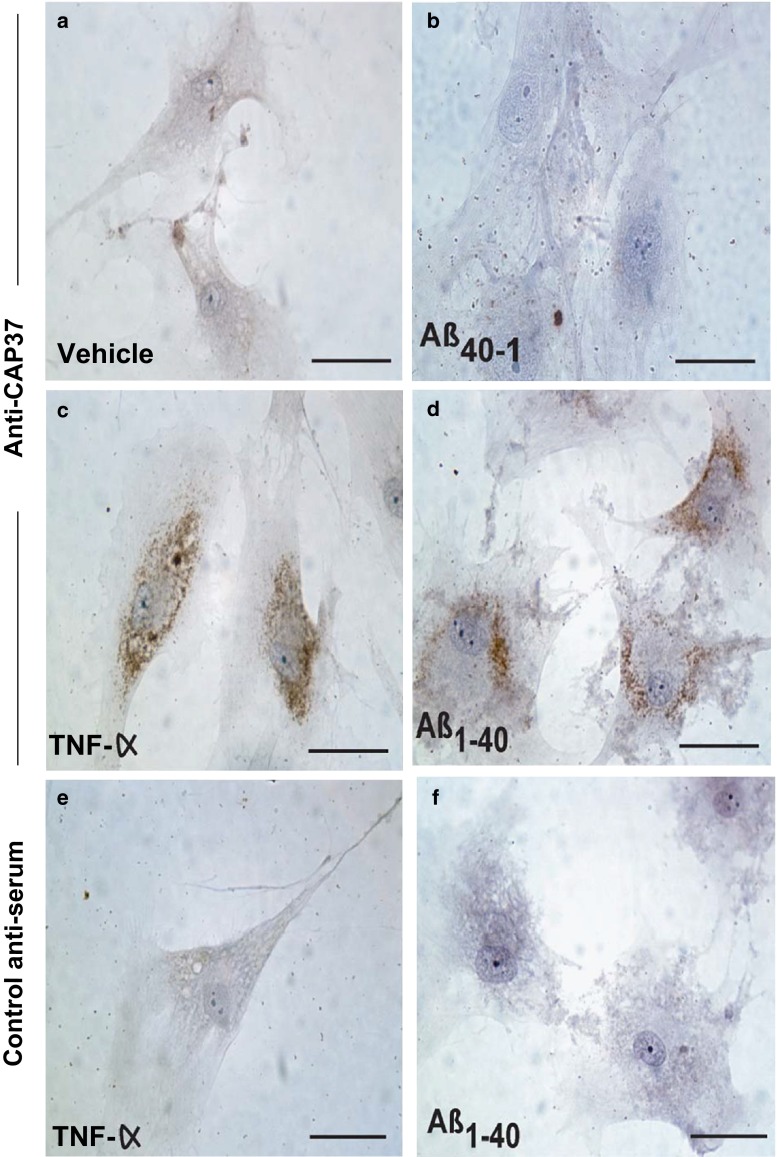
CAP37 is induced in HCN-1A primary human neurons. **a** HCN-1A neurons incubated with vehicle only (basal medium ± equivalent volume of 10 % acetic acid used as solvent for peptide). **b** HCN-1A neurons treated with Aβ_40−1_ (reverse/inactive peptide) stained with rabbit anti-CAP37 serum. **c** HCN-1A cortical neurons treated with TNF-α (25 ng/ml) and stained with rabbit anti-CAP37 serum. **d** HCN-1A cells treated with Aβ_1−40_ (125 μg/ml, pre-aggregated) and stained with rabbit anti-CAP37 serum. **e** HCN-1A cortical neurons treated with TNF-α (25 ng/ml) and stained with normal rabbit control serum. **f** HCN-1A neurons treated with Aβ_1−40_ (125 μg/ml, pre-aggregated) and stained with normal rabbit control serum. Treatments were performed overnight (17 h), and cells were stained using the Vectastain ABC peroxidase system. *Scale bars* 20 μm

The ability of two AD mediators, TNF-α, a pro-inflammatory cytokine, and Aβ_1−40_ to induce the expression of CAP37 was tested using primary human cortical neurons (HCN-1A) as a model for induced expression. These cells are non-malignant and retain their native neuronal phenotype. They have the ability to divide more rapidly due to their derivation from a patient with unilateral megalencephaly, a low-grade proliferation and migration disorder affecting neurons. We initially examined these cells for constitutive expression of CAP37 and detected faint CAP37 expression (data not shown). Faint immunopositivity was also seen in vehicle-treated controls (Fig. 5a). HCN-1A neurons were treated with TNF-α and Aβ_1−40_. Then, CAP37 expression was determined using IHC. Both TNF-α and Aβ_1−40_ induced CAP37 expression in the neuronal cytoplasm (Fig. 5c, d). Cells treated with the inactive form of Aβ (Aβ_40−1_) were not induced to express CAP37 (Fig. 5b). Minimal staining was observed with Aβ- and TNF-α-treated cells probed with rabbit control serum (Fig. 5e, f), confirming the specificity of the CAP37 staining.

### CAP37 mRNA is expressed in neurons, astrocytes, and microglia

**Fig. 6:**
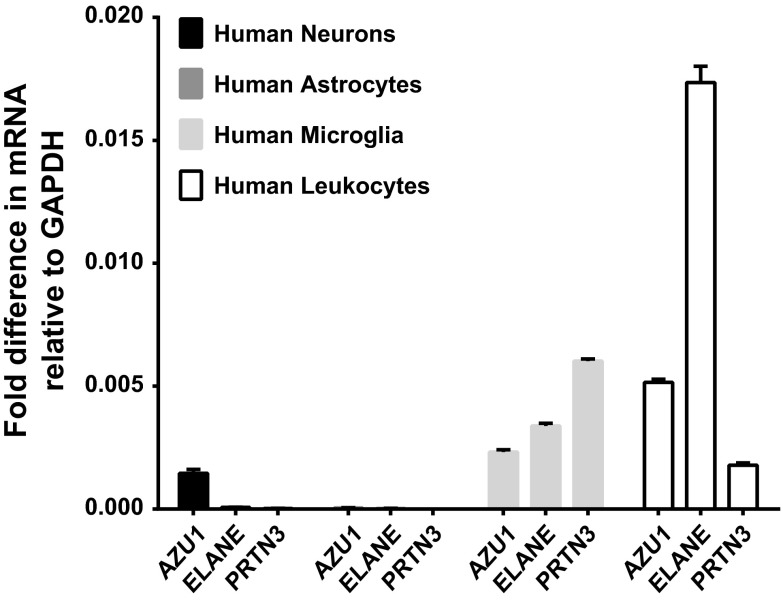
CAP37 mRNA is expressed in human primary neurons, astrocytes, microglia, and leukocytes. *AZU1*, *ELANE*, and *PRTN3* (encode for CAP37, elastase, and proteinase-3 proteins, respectively) mRNA expression in neurons (*black bars*), astrocytes (*gray bars*), microglia (*light gray bars*), and leukocytes (*open bars*) was determined using qRT-PCR. All values were normalized to *GAPDH* (internal control), and results are expressed as fold differences in mRNAs relative to *GAPDH* (2^(−ΔCT)^). Analysis of *AZU1*, *ELANE*, and *PRTN3* neuronal and glial expression was performed in triplicate, and leukocyte expression was performed in duplicate. Data are mean ± SEM of results

The expression of CAP37 mRNA in primary human neurons, astrocytes, and microglia was analyzed using qRT-PCR. *AZU1* mRNA, which encodes for CAP37 protein, was detected in resting neurons, astrocytes, and microglia (Fig. 6). *AZU1* expression in astrocytes, however, was very low compared to expression in neurons and microglia. *ELANE* (elastase) and *PRTN3* (proteinase-3), two other proteins expressed abundantly in neutrophils with high sequence homology to CAP37, also showed mRNA expression in neurons, but at considerably lower levels compared with *AZU1* (Fig. 6). *ELANE* and *PRTN3* expression was comparable to *AZU1* expression in astrocytes and microglia. To compare the levels of *AZU1*, *ELANE*, and *PRTN3* mRNA expressed in resting neurons and glial cells relative to resting leukocytes, including PMNs, we performed qRT-PCR on leukocyte cDNA isolated from buffy coats, the blood fractions containing leukocytes and platelets. Upon calculating fold differences in mRNA relative to *GAPDH,* we detected approximately five times the amount of *AZU1* mRNA, 283 times the amount of *ELANE* mRNA, and 65 times the amount of *PRTN3* mRNA in total leukocytes compared with neurons (Fig. 6). There was ~100 times the amount of *AZU1* mRNA, ~500 times the amount of *ELANE* mRNA, and ~850 times the amount of *PRNT3* mRNA in total leukocytes compared with astrocytes. We detected ~2 times the amount of *AZU1* mRNA and ~4 times the amount of *ELANE* mRNA in leukocytes compared to microglia. Interestingly, *PRTN3* expression in microglia was ~4 times higher than in total leukocytes. The C_t_, Δ C_t_, and fold difference expression values of all transcripts are shown in Table 1.

**Table 1:** Expression of *AZU1, ELANE,* and *PRTN3* transcripts in primary human neurons, astrocytes, microglia, and leukocytes

	GAPDH	AZU1			ELANE			PRTN3		
	Mean *C*_t_	*C*_t_	Δ *C*_t_	2^(−Δ *C*t)^	*C*_t_	Δ *C*_t_	2^(−Δ *C*t)^	*C*_t_	Δ *C*_t_	2^(−Δ *C*t)^
HN	17.95	27.83 27.19 27.05	9.88 9.24 9.10	0.00106 0.00165 0.00182	31.19 32.03 32.04	13.24 14.08 14.09	0.00010 5.7742 × 10^−5^ 5.7344 × 10^−5^	33.64 32.52 33.55	15.69 14.57 15.60	1.8916 × 10^−5^ 4.1114 × 10^−5^ 2.0134 × 10^−5^
	18.04	28.30 27.21 27.32	10.26 9.17 9.28	0.00817 0.00174 0.00161	33.01 31.70 31.52	14.97 13.66 13.48	3.1231 × 10^−5^ 7.7434 × 10^−5^ 8.7724 × 10^−5^			
HA	15.76	30.07 30.28 29.71	14.31 14.52 13.95	4.9119 × 10^−5^ 4.2466 × 10^−5^ 6.3042 × 10^−5^	31.41 31.28 30.60	15.65 15.52 14.84	1.9403 × 10^−5^ 2.1233 × 10^−5^ 3.4018 × 10^−5^	34.27 35.67 35.27	18.51 19.91 19.51	2.6726 × 10^−6^ 1.0127 × 10^−6^ 1.3363 × 10^−6^
	15.91	30.85 29.79	14.94 13.88	3.1814 × 10^−5^ 6.6329 × 10^−5^						
HM	14.74	23.59 23.63 23.57	8.85 8.89 8.83	0.00217 0.00211 0.00220	23.00 22.86 23.00	8.26 8.12 8.26	0.00327 0.00360 0.00327	22.11 22.09 22.16	7.37 7.35 7.42	0.00606 0.00614 0.00585
	15.37	24.01 23.95	8.65 8.59	0.00250 0.00260						
HL	13.60	21.23 21.16	7.64 7.57	0.00503 0.00528	19.50 19.39	5.91 5.80	0.01669 0.01801	22.80 22.65	9.21 9.06	0.00169 0.00188

*HN* Human neurons; *HA* human astrocytes; *HM* human microglia; *HL* human leukocytes

### Only the temporal lobe has increased expression of both CAP37 mRNA and protein during AD

**Fig. 7:**
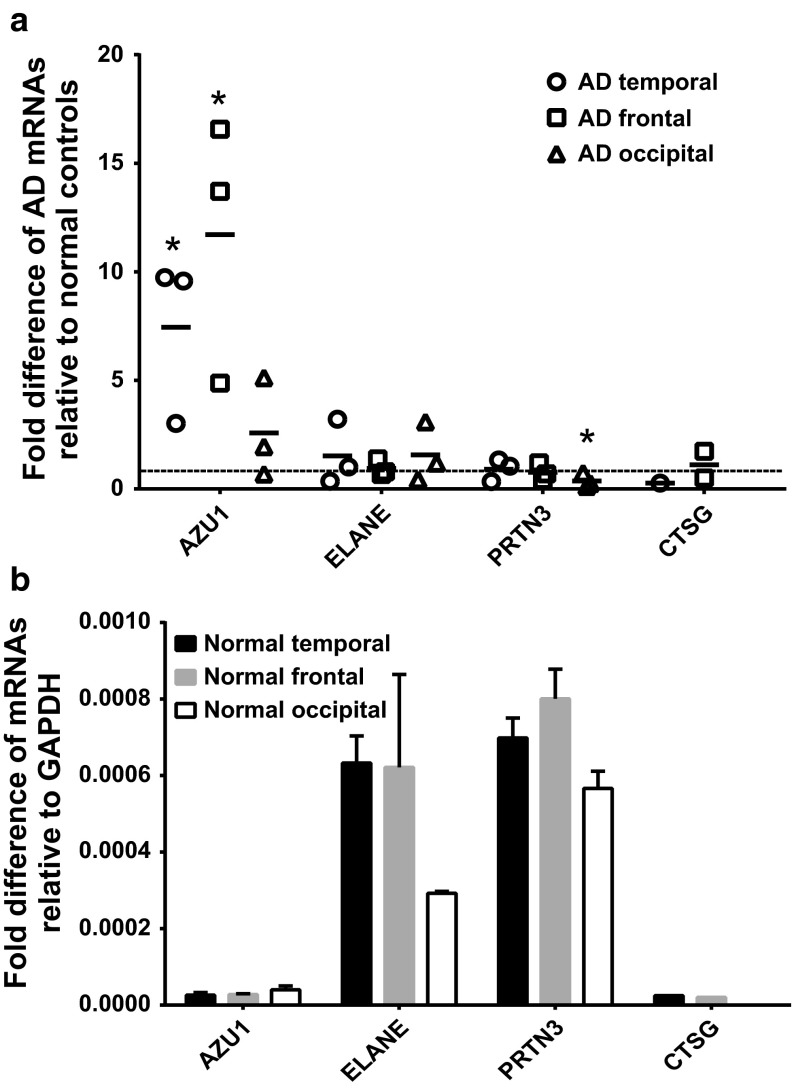
CAP37 mRNA is upregulated in AD temporal and frontal lobes. *AZU1*, *ELANE*, *PRTN3*, and *CTSG* mRNA (encode for CAP37, elastase, proteinase-3, and cathepsin G-proteins, respectively) expression was determined by performing qRT-PCR. **a** Total RNA from tissues from the temporal (*circles*, *n* = 3), frontal (*squares*, *n* = 3), and occipital lobes (*triangles*, *n* = 3) of individual AD patients (*n* = 6) was used. Values are relative to the calculated values of the normal controls for each respective tissue that were each set to 1 (indicated by the *dashed line*). **b** Total RNA from the temporal (*black bars*), frontal (*gray bars*), or occipital lobes (*open bars*) of normal adult human controls (five donor pool) expressed as fold differences in mRNAs relative to *GAPDH* (2^(−ΔCT)^). All values in **a** and **b** were normalized to *GAPDH* (internal control). Data are mean ± SEM of results. *p < 0.05 (Student’s unpaired *t* test)

Brain regions are differentially affected during AD progression. The temporal and frontal lobes are heavily impacted regions in AD, while the occipital lobe is less severely impacted. If CAP37 contributes to inflammation during AD, then temporal or frontal lobes are expected to have increased expression of CAP37 versus the occipital lobe in AD patients compared to normal controls. The mRNA expression of *AZU1*, *ELANE*, and *PRTN3* in these three lobes was compared in AD and normal controls. *CTSG* mRNA levels (cathepsin G) in the temporal and frontal lobes were also compared. Cathepsin G was included in this analysis as this protein is expressed abundantly in neutrophils and shares homology with CAP37. Significantly increased levels of *AZU1* mRNA were found in the temporal and frontal lobes of patients with AD compared with normal controls (Fig. 7a), which is consistent with these regions being heavily impacted in AD. Importantly, the mRNA levels of *ELANE*, *PRTN3*, and *CTSG* were unchanged in the temporal and frontal lobes of patients with AD compared with normal controls. No significant increase in *AZU1* expression was detected in the occipital lobe, which is a region of the brain that is less severely impacted in AD. Interestingly, *PRTN3* mRNA expression was significantly decreased in the occipital lobe of the brain with AD (Fig. 7a). Neither the cause nor the consequence of this decrease is known. Comparing the mRNA levels of *AZU1*, *ELANE*, *PRTN3*, and *CTSG* in five donor pools of normal controls, we found that levels of *AZU1* and *CTSG* were ~5–35 times lower than the levels of *ELANE* and *PRTN3* in all brain regions analyzed (Fig. 7b). As noted in the methods section, all mRNA values were normalized to *GAPDH* expression.

**Fig. 8:**
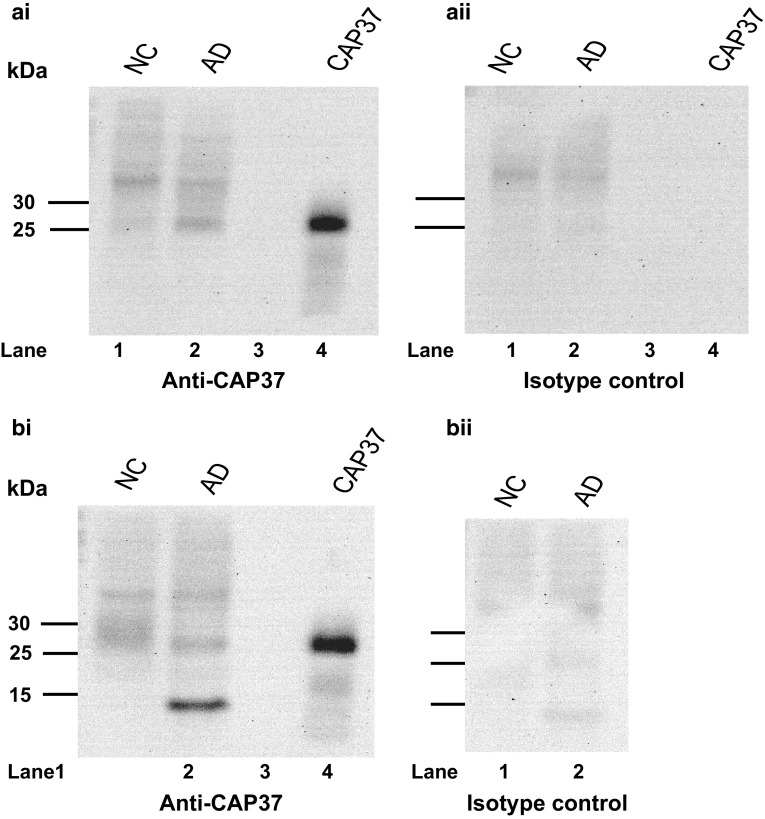
CAP37 protein expression is increased in the temporal lobes of an AD patient. Protein lysates (40 μg) from an AD patient and normal control (NC) were electrophoresed with 12.5 % SDS-PAGE gels and transferred to nitrocellulose membranes. Lane order is as follows: *Lane 1*, NC; *lane 2*, AD sample; *lane 3*, empty; *lane 4*, purified CAP37. Temporal lobe blots were probed with **ai** monoclonal anti-CAP37 (D5F10) or **aii** mouse isotype control. CAP37 migrates at 29 kDa in the AD sample on SDS-PAGE gels. CAP37 varies between 28 and 39 kDa depending on its glycosylation state. The mouse isotype control shows no corresponding 29-kDa band. Frontal lobe blots were probed with **bi** D5F10 or **bii** mouse isotype control. A 29-kDa band corresponding to the CAP37 molecular weight was observed in both the normal control and the AD patient with equivalent intensity. A dense band at ~15 kDa was observed in the AD patient lysate, but not in the normal control. Mouse isotype control shows no corresponding 29- or 15-kDa bands

CAP37 protein expression in the temporal and frontal lobes of patients with AD and normal controls was analyzed by Western blot to confirm transcript expression analysis. Total lysates from the temporal and frontal lobes showed low levels of CAP37 expression in normal controls (Fig. 8ai, bi, lane 1) as determined by the detection of an approximately 29-kDa band when blots were probed with monoclonal anti-CAP37. This is the same molecular weight to which the isolated native CAP37 migrated (Fig. 8ai, bi, lane 4). The tissues from the patient with AD demonstrated a band of increased intensity in the temporal lobe lysate that was absent in the normal control (Fig. 8ai, lane 1, 2). This result confirms qRT-PCR and IHC results showing an increase in CAP37 expression in the temporal lobe of AD patients. We observed no difference in the intensity of the 29-kDa CAP37 band in the frontal lobe normal control or AD lysates (Fig. 8bi, lanes 1, 2). However, the extract from the frontal lobe of the patient with AD demonstrated a band of high intensity migrating at a molecular weight of ~15 kDa (Fig. 8bi, lane 2). This was not observed in the normal control (Fig. 8bi, lane 1). Blots were also probed with mouse isotype control which showed no corresponding bands at 29 or 15 kDa for the total lysates or purified CAP37 (Fig. 8aii, bii, lanes 1, 2, 4).

### CAP37 protein is not detected in astrocytes or microglia by Western blotting

**Fig. 9:**
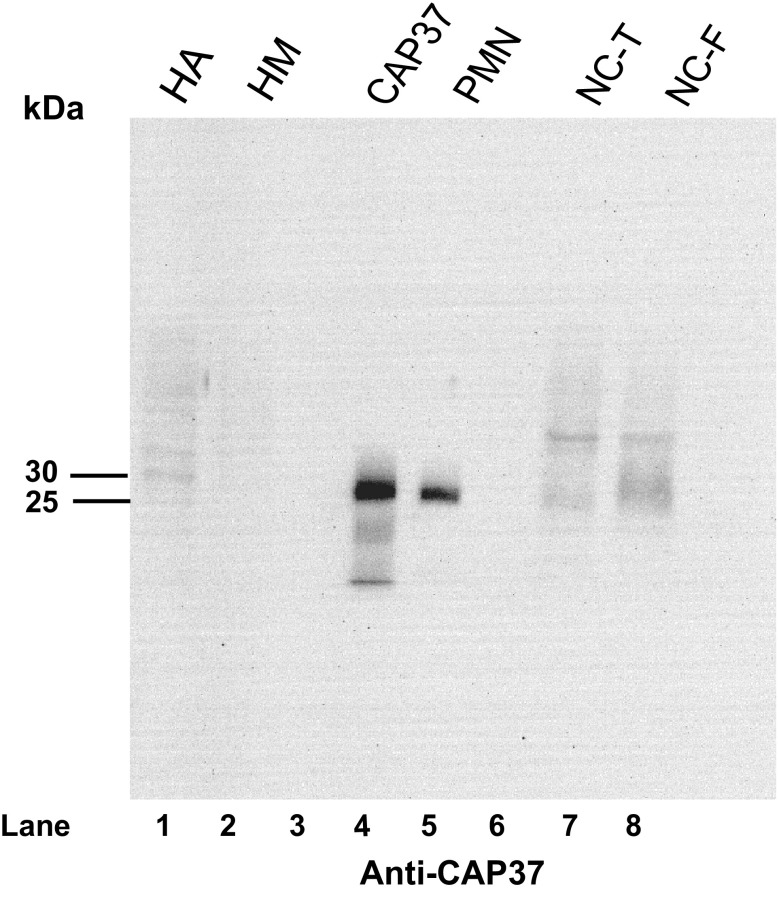
CAP37 protein is not detected in astrocytes or microglia by Western blotting. Protein lysates in the following order were loaded: *lane 1*, human astrocytes (HA, 40 μg); *lane 2*, human microglia (HM, 40 μg); *lane 3*, empty; *lane 4*, purified CAP37 (5 ng); *lane 5*, PMNs (50 ng); *lane 6*, empty; *lane 7*, normal control brain lysates (40 μg) from the temporal lobe (NC-T); *lane 8*, normal control brain lysates from the frontal lobe (NC-F) and were electrophoresed with 12.5 % SDS-PAGE gels and transferred to nitrocellulose membranes. Blot was probed with anti-CAP37 (D5F10). No band corresponding to the CAP37 molecular weight was observed in HA or HM lysates

Since we observed CAP37 transcript in astrocytes and microglia, but did not observe CAP37 protein in these cells by IHC analysis of brain sections, we performed Western blotting on proteins extracted from astrocytes and microglial cells. No band corresponding to the CAP37 molecular weight was detected in astrocytes or microglia (Fig. 9, lanes 1,2). Purified native CAP37, PMN lysate, and total lysates from the temporal and frontal lobes of normal controls were loaded as controls for CAP37 migration and expression. Bands of high intensity can be seen for isolated CAP37 (Fig. 9, lane 4) and PMN lysates (Fig. 9, lane 5) between 25 and 30 kDa. Low levels of CAP37 were observed in temporal and frontal lobes of normal controls (Fig. 9, lanes 7, 8).

## Discussion

The common phenotype among many neurodegenerative diseases is the death of neurons, which leads to cognitive decline in patients. Therapeutic routes that clinicians and researchers focus on include preventing neuron death, identifying methods to reverse or treat the neuronal damage, or using stem cell therapy to replace the defunct neurons with new healthy neurons. Some researchers focus on a prophylactic approach, which involves identifying early disease biomarkers. Such biomarkers could then be used to determine treatment options before clinical symptoms arise. Further research identifying and characterizing these biomarkers could potentially lead to therapeutics that prevent disease progression.

Unexpectedly, hippocampal staining with IHC herein demonstrated CAP37 expression in brain tissue from two controls that contained mild-to-moderate AD pathological changes, without showing clinical symptoms of dementia. This surprising finding indicates the potential use of this early increase in CAP37 expression as an early AD biomarker. Changes in protein levels in cerebrospinal fluid (CSF) have recently been demonstrated in forms of dementia, such as AD, and CSF is now considered a key source for identifying biomarkers that predict the onset of dementia (Sun et al. 2003; Kaerst et al. 2013; Kapaki et al. 2013; Scherling et al. 2014). Interestingly, CAP37 has been reported in the CSF at significantly increased levels in patients with bacterial meningitis (Linder et al. 2011). Whether there is an increase or decrease in CAP37 expression in the CSF of AD patients is currently unknown, but it is worth investigating in future studies. We must be able to identify more of these biomarkers that can be detected at early stages of these progressive diseases to determine which are toxic and can be targeted, and which are protective and can potentially be developed into therapeutics.

Within the past 5 years, researchers have postulated that chronic bacterial and viral infections may be responsible for initiating the formation of Aβ plaques and thus the subsequent pathological events that occur in AD (Balin et al. 2008; Miklossy 2008; Urosevic and Martins 2008; Bu et al. 2014; Piacentini et al. 2014; Welling et al. 2014). The role of antimicrobial peptides (AMPs) which increase in response to these infections has also been questioned. Aβ itself has been determined to be an AMP and has also been found to inhibit both the H3N2 and H1N1 influenza A viruses (Soscia et al. 2010; White et al. 2014). As previously mentioned, β-defensin-1 is another AMP that is upregulated in AD (Williams et al. 2013). CAP37 can now also be added to this list. Many pathogens that cause chronic infections have been found to compromise the blood–brain barrier which is disrupted in AD (Dickstein et al. 2006; van Sorge and Doran 2012; Erickson and Banks 2013; Marques et al. 2013; White et al. 2014). Notably, CAP37 promotes vascular permeability by inducing rearrangement of the cytoskeleton in endothelial cells and increasing endothelial cell permeability (Gautam et al. 2001; Ley 2001). One study has suggested that CAP37 may be involved in the breakdown of the blood–retinal barrier (Skondra et al. 2008), but direct effects of CAP37 on the blood–brain barrier are currently unknown.

In the present study, we showed that CAP37 is the only neutrophil-derived mRNA of the four analyzed homologs that demonstrates high expression in neurons and increased levels in the AD brain. Although IHC analysis and Western blotting did not reveal CAP37 expression in astrocytes or microglia, we did detect CAP37 transcript in these cells. CAP37, therefore, could potentially be translated and expressed in microglia and astrocytes in other conditions or perhaps pathologies with more acute inflammation. Further studies must be conducted to determine this. The lack of an increase in the neutrophil markers *ELANE*, *PRTN3*, and *CTSG* (Korkmaz et al. 2010) in patients with AD indicates that the increase in *AZU1* (CAP37) expression was not due to an increase in neutrophil influx into the brains of these patients. The low expression of *ELANE*, *PRTN3*, and *CTSG* in neurons and the high expression of these transcripts in brain tissues of normal controls indicate the source of these mRNAs is non-neuronal brain cells such as neutrophils or glial cells.

During AD, the entorhinal cortex is the first region of the brain that is affected, and it acts as a gateway for damage into the hippocampus, where atrophy has been demonstrated in many presymptomatic individuals (Fox et al. 1996; Scahill et al. 2002). As the disease progresses, atrophy begins to occur in the inferior temporal cortex, cingulate cortex, and the precuneus, a region of the superior parietal lobe. In advanced stages of the disease, atrophy of the frontal lobe occurs before eventual spread to the entire neocortex (Scahill et al. 2002; Serrano-Pozo et al. 2011; Khan et al. 2014). Regions of the neocortex that suffer the most severe atrophy include the inferior temporal cortex and the prefrontal cortex, while the occipital cortex is one of the last and least severely impacted (Serrano-Pozo et al. 2011). Herein, we detected CAP37 within pyramidal neuron cell bodies in cortical layers 3 and 5 of the temporal and parietal lobes, which are the specific layers with the most neurofibrillary degeneration in AD (Serrano-Pozo et al. 2011). The levels of CAP37 in neurons of the hippocampus were already elevated in age-matched controls. We did not observe an increase in CAP37 in neurons from this location in patients with AD. Our observation that tissues from the age-matched controls contained a substantial number of plaques and some neurofibrillary tangles indicates that CAP37 may be an early factor in the disease process. These age-matched control tissues demonstrated pathological changes, but the patients did not show clinical symptoms of dementia. However, based on the progressive nature of AD, there is substantial potential for such clinical symptoms to have developed with increased longevity of the patients. The high increase in CAP37 mRNA and the lack of an increase in CAP37 protein in the frontal lobe suggest that CAP37 may also precede the pattern of atrophy that occurs in AD. We cannot, however, rule out the possibility that the 15-kDa band observed in the frontal lobe of the patient with AD is a proteolytic degradation product of CAP37 that is only present in the tissues from the patient with AD and not the control. Many Aβ degradation products cleaved by enzymes, such as neprilysin and insulin-degrading enzyme, have recently been identified; the role of several altered proteolytic pathways in AD is under investigation (De Strooper 2010; Saido and Leissring 2012). Whether CAP37 is also degraded or cleaved in particular regions of the brain in AD is currently unknown. The increase in CAP37 in regions severely impacted in AD suggests that CAP37 may play a role in regulating the toxic events that occur in the specific areas of the brain that suffer the greatest atrophy in AD.

Although we are uncertain whether CAP37 expression induces molecular events that cause AD progression or is a result of molecular events that arise in AD, our observations indicate that CAP37 does not directly overlap with Aβ or tau. The plaques and tangles could spread into CAP37-positive regions (or vice versa) with disease progression. Our results using HCN-1A neurons showed that Aβ_1−40_ could induce CAP37 expression, while the inactive peptide Aβ_40−1_ could not. This finding indicates that CAP37 induction is specific to the Aβ structure associated with AD and not to a similar protein sequence found within the inactive peptide. These results also suggest that CAP37 expression may occur after Aβ accumulation. If so, CAP37 would also fall into the microglial activation and inflammatory response stage of the amyloid cascade hypothesis. The amyloid cascade hypothesis posits that AD pathological events are initiated by Aβ, which augments an inflammatory response that leads to oxidative stress, and is followed by tangles and widespread neuron degeneration (Citron 2004, 2010; Harrington 2012; McGeer and McGeer 2013). Most ongoing clinical trials are understandably aimed at targeting Aβ in an attempt to stop cascade initiation. Aβ, which may be responsible for many toxic AD events, has a plethora of receptors it can bind and activate (Doens and Fernandez 2014). Blocking Aβ, therefore, may block a large number of cellular events that are needed to maintain homeostasis and thus may produce unintended effects. Although anti-inflammatory approaches may not be the central focus for AD therapeutics, it is plausible that targeting specific molecules responsible for initiating fewer signaling pathways in this component of the cascade may decrease nonspecific events and adverse side effects.

Inflammation in the brain is predominantly driven by resident macrophages known as microglia. These cells function primarily to survey the microenvironment for pathogens, maintain neuronal synaptic integrity, and kill exogenous pathogens. Alterations or disruptions in the homeostasis of the brain are known to activate the microglia, causing them to change from a ramified to ameboid morphology, upregulate specific cell surface receptors, and secrete toxic molecules, including pro-inflammatory cytokines, nitric oxide (NO), and reactive oxygen species (ROS) (Perry et al. 2010). Importantly, CAP37 activates microglial cells by various mechanisms including the ability to change the morphology of microglia from ramified to ameboid, induce the release of pro-inflammatory cytokines, increase the expression of class II major histocompatibility antigens and chemokines, and induce phagocytic and chemotactic activities (Pereira et al. 2003). The receptor for CAP37 on microglial cells is currently unknown. However, previous studies from our laboratory suggest that signaling may occur through a G-protein-coupled receptor since CAP37 induced chemotaxis of human corneal epithelial cells is inhibited with pertussis toxin, a known disruptor of GPCR activation (Griffith et al. 2013).

## Conclusions

Based on the results of the present study, we infer that CAP37, an established inflammatory mediator previously shown to activate microglial cells, may mediate the chronic neuroinflammation associated with AD from within the brain parenchyma. Whether CAP37 could be a potential target or therapeutic is uncertain. However, our data suggest that CAP37 is involved in the AD process, and it should be a strong candidate for further investigation.

## Abbreviations

AD: Alzheimer’s disease
CAP37: Cationic antimicrobial protein of 37 kDa
PMN: Polymorphonuclear leukocyte
IHC: Immunohistochemistry
TNF-α: Tumor necrosis factor-alpha
Aβ: Amyloid-beta
qRT-PCR: Quantitative reverse transcription polymerase chain reaction
PKC: Protein kinase C
LPS: Lipopolysaccharide
CNS: Central nervous system
CERAD: Consortium to establish a registry for Alzheimer’s disease
DMEM: Dulbecco’s modified Eagle’s medium
ATCC: American type culture collection
HRP: Horseradish peroxidase
GAPDH: Glyceraldehyde dehydrogenase
BCA: Bicinchoninic acid
TBST: Tris-buffered saline with Tween
ECL: Enhanced chemiluminescence
CA: *Cornu Ammonis*
CSF: Cerebrospinal fluid
AMP: Antimicrobial peptide
NO: Nitric oxide
ROS: Reactive oxygen species
